# Morphogenesis and mechanical properties of *Bacillus amyloliquefaciens* biofilms: a comparative study of rough and smooth morphotypes

**DOI:** 10.1016/j.crmicr.2025.100403

**Published:** 2025-05-10

**Authors:** Emmanuelle Baudu, Eric Raspaud, Catherine Fontagné-Faucher, Yassine Nait Chabane, Claire-Emmanuelle Marcato-Romain

**Affiliations:** a IUT de Toulouse Auch Castres, LBAE URU 4565 (Laboratoire de Biotechnologies Agroalimentaire et Environnementale), Université de Toulouse, 24 rue d'Embaquès, Auch F-32000, France; b CNRS, Laboratoire de Physique des Solides, Université Paris-Saclay, Orsay 91405, France

**Keywords:** Pellicle, Colony expansion, Mechanical properties, Stiffness, Elasticity

## Abstract

*Bacillus amyloliquefaciens*, a Gram-positive, endospore-forming bacterium, is ubiquitously found in diverse environments. Its resilience in harsh conditions and biofilm-forming capabilities make it a promising candidate for various agricultural applications. *B. amyloliquefaciens* can also colonize the air-liquid interface by producing pellicles. Despite its potential, the characterization of *B. amyloliquefaciens* biofilms remains underexplored. We investigated colony expansion and pellicle formation kinetic, followed by an analysis of the mechanical properties of the latter. This work focused on *B. amyloliquefaciens* strain L-17, which produced two morphotypes on agar plate: 1) a **rough** (R) morphotype exhibiting quick colony expansion and 2) a **smooth** (S) morphotype with a slower but extended colony expansion. The morphogenesis model of pellicle formation proposed in the literature for *Bacillus subtilis* was applicable to *B. amyloliquefaciens*, with few modifications for the smooth morphotype, which needed a longer period to spread over the surface. Mature pellicles were visually homogeneous and heterogeneous for R and S, respectively. Surprisingly, (i) their deformability by stretching was heterogeneous for the former and homogeneous for the latter; (ii) R produced a stiff pellicle whereas the entire smooth pellicle showed an elastic response.

Taken together, results of this study confirmed that the existence of two distinct morphotypes within the same strain offers a unique opportunity to study the diversity of behaviors in *B. amyloliquefaciens*, providing insights into its potential applications in agriculture and beyond. Moreover, they led to the hypothesis that surfactant production and flagella expression are differentiating factors between both morphotypes.

## Highlights

• One strain can form two completely different pellicles.
• The rough morphotype produced a pellicle 5 times stiffer than the smooth.
• The smooth pellicle showed a spatially homogeneous elasticity.
• Rough colonies expansion was faster than the smooth one.

## Graphical abstract

**Figure:**
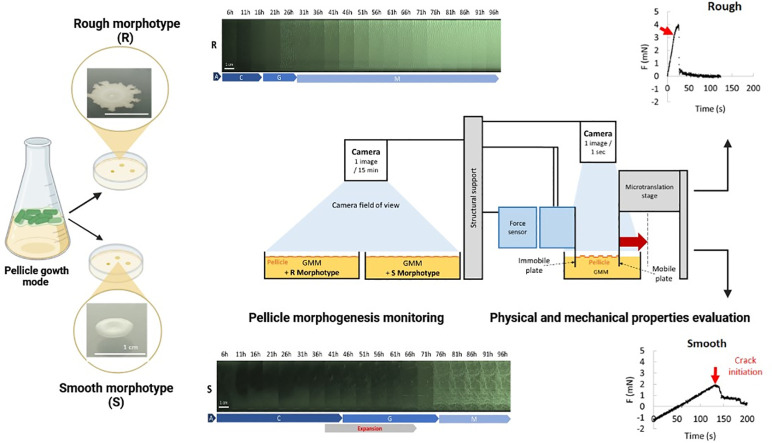

## 1 Introduction

*Bacillus amyloliquefaciens*, ubiquitously found in food, plants, animals, soil and in different environments, belongs to the operational group *Bacillus amyloliquefaciens* (OGBa), all of which are Gram-positive, endospore-forming and rod-shaped bacteria (Ngalimat et al., 2021; WoldemariamYohannes et al., 2020). Its wide existence in a variety of harsh environments and endospore-forming capabilities enable it to withstand extreme conditions and persist in the environment. Recently, members of OGBa have emerged as an interesting source of biocontrol agents for the management of pathogenic fungi, biofertilizer potential and stress tolerance enhancer for various crops in agriculture (Gautam et al., 2019; Luo et al., 2022; Zalila-Kolsi et al., 2023). *B. amyloliquefaciens* is a plant growth-promoting rhizobacterium (PGPR) renowned for its robust biofilm formation across diverse ecosystems (Jimoh et al., 2023; Kasim et al., 2016; Zaidi-Ait Salem et al., 2021b). Several strains, such as E4CDX2 and ATB-BAS010, have been commercialized as fertilizers. Recently, its ability to stabilize soil aggregates has been suggested (Deka et al., 2019). However, the characterization of *B. amyloliquefaciens* biofilms remains insufficient to fully understand their impact on soil aggregates.

Biofilms are structured communities of microbial cells that develop at interfaces and are encased in a self-produced matrix of extracellular polymeric substances (EPS), such as exopolysaccharides, nucleic acids (eDNA and eRNA), proteins and lipids, and others (Arnaouteli et al., 2023; Flemming and Wingender, 2010; Karygianni et al., 2020). Study of biofilms, particularly those formed by the *Bacillus* genus, has garnered significant attention due to their unique properties and potential application (Coleri Cihan et al., 2017; Sowmiya et al., 2024). In the *Bacillus subtilis* group, the matrix is mainly composed of polysaccharides mainly synthesized by the gene products of the *epsA-O* operon, the *tapA-sipW-tasA* operon for the amyloid-like protein TasA and the amphiphilic protein BslA (Sanchez-Vizuete et al., 2022). Similar genes have been found in *B. amyloliquefaciens* genome (Morris et al., 2017; Xu et al., 2012; Yang et al., 2015), however, the biochemical composition of the matrix remains much less well described than in *B. subtilis*.

Besides its ability to form biofilms on solid biotic or abiotic supports, *B. amyloliquefaciens* can colonize the air-liquid interface, thus forming a special biofilm often referred to as “pellicle” that exhibit remarkable mechanical properties (Kovács et al., 2012; Xu et al., 2023; Zaidi-Ait Salem et al., 2021b). Pellicle formation is a dynamic process involving bacterial multiplication, differentiation and production of an extracellular matrix, leading to a complex multicellular community (Douarche et al., 2015; Nait Chabane et al., 2014; Rajitha et al., 2020). The process of establishment of this specific type of floating biofilm requires the presence of oxygen and certain cations, such as Ca^2+^, Mn^2+^, Cu^2+^ and Zn^2+^, which are essential for the stability and maturation of the pellicle (Dubay et al., 2022; Fall et al., 2006). In *B. subtilis*, the formation of pellicles is a multi-step process that includes initial attachment, monolayer development and maturation into a three-dimensional structure. Kobayashi (2007) and Lee et al. (2019) have detailed the stages of pellicle formation in *B. subtilis*, but this process has yet to be observed in *B. amyloliquefaciens*. Kearns and Losick (2003) reported that biofilm formation studies in ancestral strains of *B. subtilis* revealed significant impairment in cell motility due to the domestication of commonly used laboratory derivatives. The intricate relationship between motility and biofilm formation has garnered renewed interest. For solid-supported biofilms, flagellar-mediated motility, such as swimming and swarming, appears to be inversely regulated with biofilm formation, whereas gliding motility and biofilm formation seem to occur under similar conditions. In *B. subtilis*, pellicle formation has been shown to be triggered by oxygen depletion in the liquid medium (Hölscher et al., 2015; Sanchez-Vizuete et al., 2022). Upon detecting this depletion, motile cells migrate towards the air-liquid interface. Mutations in motility genes can delay pellicle formation without altering its final appearance (Hölscher et al., 2015). In *Shewanella oneidensis*, the loss of motility affects pellicle formation but not surface-associated biofilms, highlighting the critical role of motility in pellicle formation for this species (Liang et al., 2010). Therefore, incorporating cell motility as an initial step in pellicle formation is of significant interest.

Finally, to gain a deeper understanding of the mechanisms behind the establishment of this type of biofilm, it is crucial to investigate its mechanical characteristics. In fact, Hollenbeck et al. (2016) observed that *B. subtilis* pellicle behaved viscoelastically when it endured natural compression due to growth in a confined space and viscoplastically under large deformations such as an applied tension. In their study on *B. subtilis*, Krajnc et al. (2022) established a link between biofilm development at the air-liquid interface and the spatial distribution of bacterial cells over the thickness of the pellicle, as well as cell viability. The authors also showed a correlation between mechanical properties and morphological changes as well as aggregation state, filament formation, biofilm thickness and spore formation in the growing pellicle.

The studied strain was *B. amyloliquefaciens* L-17, a wild-type strain isolated in the West of France and that showed interesting capacities for pellicle production (Zaidi-Ait Salem et al., 2021a, 2021b). The L-17 strain exhibits two distinct colony morphotypes: rough and smooth. This phenomenon is expected, given the *Bacillus* genus's well-documented ability to swiftly adapt to environmental changes (Losick, 2020; Saxena et al., 2020). The rough morphotype is able to form a cohesive and hydrophobic biofilm at the air-liquid interface and has already been described by Zaidi-Ait Salem et al. (2021b), but the smooth morphotype has not yet been described for this strain. Both morphotypes have been compared in this study.

Here, we have, for the first time, detailed the stages of biofilm formation at the air-liquid interface in *B. amyloliquefaciens* and explored its mechanical characteristics. This work has allowed us to establish a link between cell motility and the initial stages of biofilm formation. We then connected this dynamic analysis with the mechanical properties of the pellicles. Through this research, we aim to elucidate the complex mechanisms underlying the formation and resilience of floating biofilms, thereby enhancing our understanding of bacterial survival strategies and the potential applications of this bacterium and its biofilms in various fields.

## 2 Material and method

### 2.1 *B. amyloliquefaciens* L-17 strain and growth conditions

*B. amyloliquefaciens* L-17 was isolated by the Laboratoire de Biotechnologies Agroalimentaire et Environnementale (culture collection WDCM 1016, *B. amyloliquefaciens* Université de Toulouse, Auch, France) (Zaidi-Ait Salem et al., 2021a). This strain presented originally a rough colony on agar medium. During experiments, a smooth morphotype was isolated. In this study both morphotypes were compared in the same conditions and unless specified, the following protocols were similarly applied on both.

To ensure that the smooth morphotype was a natural phenotypic variant and not a mutant or contamination, we have carried out genomic and metabolic verifications using the BOX- A1R, (GTG)-5-PCR fingerprints and API 50 CH galleries on the variants and the reference strain *B. amyloliquefaciens* DSM 7. It resulted in similar patterns for both morphotypes and slightly different from the reference strain confirming that it was indeed the *B. amyloliquefaciens* L-17 strain (data not shown).

L-17 was conserved at −20 °C in 50 % glycerol. Prior to each experiment, 20 mL of fresh tryptic soy broth (TSB; Millipore) was inoculated with 100 µL of glycerol stock and incubated overnight at 30 °C, 130 rpm. Preliminary tests showed that *B. amyloliquefaciens* L-17 was unable to produce a thick pellicle in TSB in 24 h at 30 °C (data not shown). All following culture experiments used a medium enhancing pellicle formation: Glucose enriched Minimum Medium (GMM) or GMM agar (GMMA). GMM composition in g/L: glucose 20, TRIS-HCl 14.97; TRIS-base 0.6; NH_4_Cl 0.5; CaCl_2_ 0.05; MgSO_4_,7H_2_O 0.05; FeSO_4_,7H_2_O 0.005; MnSO_4_,H_2_O 0.0037; yeast extract 2; pH 7.

### 2.2 Colony shape and expansion on agar medium

Colonies morphology was observed on solid agar (1.5 %) GMMA. Several (*n* > 9) drops of an overnight culture were deposed on GMMA. Pictures of the colonies were taken after 24 h of incubation at 30 °C.

Expansion rate of colonies on agar plate can express a swarming or a swimming type of motility. Swimming and swarming expansion assays were done on GMMA containing 0.3 % and 0.6 % agar respectively. One drop of an overnight culture was deposed at the center of the Petri dish (*n* > 6) and dishes were incubated at 30 °C for 24 h. Colonies diameter was measured at 6 h and 24 h with ImageJ v.1.54d software (Schneider et al., 2012). Phase alternations were visually appreciated by counting concentric rings in the colony.

Statistic tests were performed by using XLStat 2023.2.1414 (Shapiro-Wilk’s test and *T*-test).

### 2.3 Monitoring pellicle morphogenesis

**Fig. 1:**
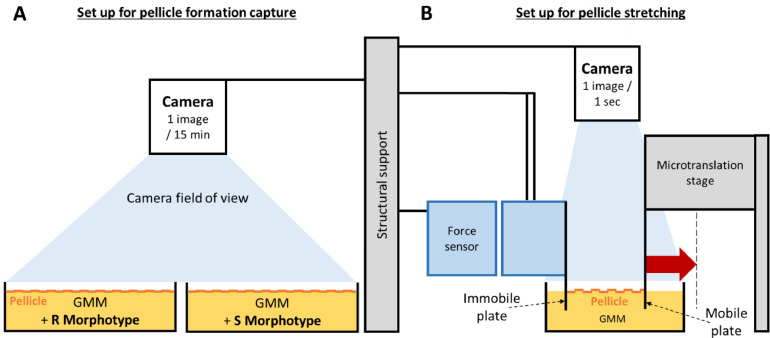
Schematic diagram of the experimental setup. (A) A high-quality camera is taking a photo every 15 min during pellicle formation (6 h – 96 h). (B) Stretching of a pellicle formed in the stretching device. Red arrow illustrates a typical displacement of the microtranslation stage. This translation induces a pellicle mechanical deformation.

Pellicles were produced in static conditions, in a sterile chamber maintained at 23 °C. Uncovered rectangular dishes (62 × 46 mm) were filled with 25 mL of GMM inoculated at a final optical density at 600 nm of 0.1 (approximatively 10^8^ CFU/mL), with no additional feeding during the incubation. A dish of non-inoculated MMG was always kept in the same conditions as a sterility control. A camera took pictures at 1 frame per 15 min from 6 h to 96 h of incubation, as represented in Fig. 1**A**. Images were annotated with the time of growth using Python 3.11 and a movie was created by stacking the pictures with ImageJ v.1.54d software.

### 2.4 Stretching device for mechanical assay

Pellicles were produced as previously described; two facing vertical 41-mm width plates (polyethylene terephthalate) were partly immerged in the medium, allowing pellicle adhesion to the plates, as described in Trejo et al. (2013) (Fig. 1**B**). One plate named “immobile plate” was connected to a force sensor and the other named “mobile plate” to a translation stage. Approximatively 13–14 mm of distance separated the plates, similarly to past experiments, in order to facilitate the results comparison. Only mature pellicles were mechanically stressed, 3 days after the inoculation for the rough morphotype and 4 days for the smooth morphotype. Before stretching, pellicle around the plates was cut with a scalpel to facilitate the analysis; in that configuration, only the pellicle’s part located between the two plates contributed to the detected forces.

The force device was first detailed in Trejo et al. (2013). Shortly, in our experiments, the mobile plate, which was connected to a motorized translation stage, moved laterally from 0 to 12 mm at a constant speed (160 µm/*sec*). During motion, pellicle was strained and consequently stressed. Resulting force values were recorded every 100 msec by a force sensor connected to the “immobile plate” (Fig. 1**B**). The force sensor corresponds to a double-cantilever appropriate to detect milliNewton (mN) forces; its stiffness is equal to 19.5 mN/mm. When pulled or pushed, the double-cantilever deflected and reached new positions that self-equilibrated the applied force. Deflection was then precisely measured through a capacitive jauge, capable of detecting precise micrometric variations at a resolution of about 0.40 μm. Interestingly, pulling and pushing forces can be simply discriminated in the device as they generate positive or negative values around a “zero-force” value. This “zero-force” value was measured in our setup after pellicle failure, when the immobile plate was not connected to the pellicle anymore.

Due to an appropriate cantilever stiffness, a 1-milliNewton force shifts the position of the “immobile plate” by 0.05 mm while, at the same time, the “mobile” plate must be moved by 0.5 to few millimeters to generate this force level within the pellicle. Although being slightly displaced, and for clarity reasons in the following text, the plate connected to the force sensor is still considered as “immobile” as opposed to the “mobile” one. Note that we have taken this slight change in the position of the “immobile” plate into account when analyzing the local deformability of pellicles and determining their relative positions.

### 2.5 Images analysis

Pellicles were not fully uniform and presented some optically visible non-uniformities that could be tracked by image processing. Color pictures were first converted into 32 bits or 8 bits images (greyscale) with ImageJ v.1.54d software. Then, displacement of inhomogeneities and emergence of periodic wrinkles were simply tracked with ImageJ v.1.54d software, by analyzing the grey level profile within selected regions of interest. Deformability analysis required a more sensitive and local method. Inhomogeneities were used as “particles” in the optical detection method named “Particle Image Velocimetry” (PIV) (PIV Analyser, 2025; PIV (Particle Image Velocimetry), 2025), a classical method to map the flow of particles (Hollenbeck et al., 2016). Here they moved upon applied relative deformation also named mechanical “strain” in mechanics. In order to detect precisely the local displacements, a pair of images was sliced into small interrogation windows (mostly 32 × 32 pixels) and in larger search windows (typically 50 × 50 pixels in our case) and grey values of each pixel were analyzed. Displacements field was then extracted by computing correlations between the greyvalues.

## 3 Results and discussion

The *B. amyloliquefaciens* L-17 strain presented originally a rough colony on agar medium (Zaidi-Ait Salem et al., 2021b). After days of experiments in the laboratory, a new smooth morphotype of the L-17 strain spontaneously appeared and was isolated. In this context, we consider pellicle as a particular biofilm because it forms at the air-liquid interface without any physical support**.** Therefore, both terms are used in this study to describe pellicle. In the following study, we examine in detail the differences in morphological traits and mechanical responses between biofilms of both morphotypes.

### 3.1 Colonies morphology and expansion on agar plates

**Fig. 2:**
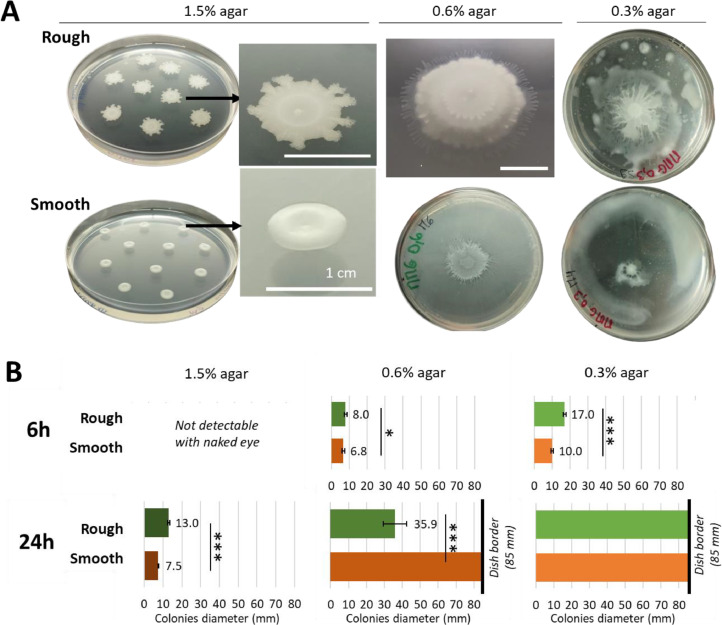
(A) *B. amyloliquefaciens* L-17 morphotypes (rough and smooth) on GMMA medium after 24 h of incubation at 30 °C. Petri dishes diameter is 85 mm. (B) Colonies diameters (mm) of both morphotypes in Petri dish depending on the agar ratio and the incubation time. At 6 h, colonies were too small to be quantified on 1.5 % agar medium. Mean ± SD. *n* > 6. *T*-test results: * = 0.05 > *p*-value > 0.01; *** = *p*-value < 0.001. Scale bar is 1 cm.

Colonies of *B. amyloliquefaciens* L-17 exhibited two contrasted morphotypes after 24 h of incubation on GMMA dishes, as shown in Fig. 2**A**. Morphological variations were evident across all agar concentrations (1.5, 0.6 or 0.3 %). Typically, 1.5 % agar plates are employed to emphasize colony morphotypes, whereas 0.6 % and 0.3 % agar plates are utilized to assess swarming and swimming capabilities, respectively. In our study, we performed both swarming and swimming assays to examine two critical morphotype features: colony morphology at 24 h (Fig. 2**A**) and expansion rate, quantified by the colony diameter at 6 and 24 h (Fig. 2**B**).

After 24 h, large (∅ = 13.0 mm ± 0.8), irregular, dry, flat and opaque colonies emerged on 1.5 % agar plates, consistent with previous observations. These morphological traits defined the common **rough** morphotype frequently observed in L-17 strain colonies. The colonies displayed a concentric ring-like morphotype with dry branches of 1–2 mm at the periphery. Conversely, some colonies of the same strain were smaller (∅ = 7.5 mm ± 0.2), shiny, moist to mucoid, convex and translucent. These colonies were homogeneous, with regular circular edges and a donut shape, a morphotype typically associated with the **smooth** morphotype.

After 6 h on 0.6 % agar, the visible horizontal expansion of **rough** colonies was significantly greater than that of the smooth morphotype. Interestingly, after 24 h on 0.6 % agar, this trend reversed, with horizontal expansion of smooth colonies significantly higher than rough colonies. Notably, the rough morphotype grew thicker rather than colonizing the entire dish, producing a substantial amount of white and opaque EPS. This vertical growth has been previously described in *B. subtilis* by Tasaki et al. (2017) as a strategy to grow on media with high agar concentration, where sliding motility is reduced compared to low agar concentration. Rough colonies still exhibited a concentric ring-like morphology. On the opposite, cells of the **smooth** morphotype swarmed to the dish borders, thus covering the whole dish with a thin layer of transparent and mucous EPS. Swarming is described as a periodic expansion process with an alternation of migration phases and consolidation phases and has been well-studied in *B. subtilis* (Kearns and Losick, 2003; Tasaki et al., 2017). Under favorable conditions, the migration phase on 0.7 % agar plates lasts approximately 2 to 3 h, followed by a consolidation phase of 3 to 5 h. Consequently, it generally takes *B. subtilis* about 1.5 days to colonize 50 mm of the dish. However, when agar concentration is reduced the ratio of the migration phase to the consolidation phase increases, allowing cells to colonize the dish more quickly (approximately 12 h to colonize a diameter of 50 mm). In this scenario, the colony is often thinner due to a reduced consolidation phase (Tasaki et al., 2017). In the present study, at least 4 phase alternations were visible in rough morphotype colonies, with an expansion area of few millimeters each. At the contrary, only 2 migration circles were visible in smooth colonies, but they were larger (centimeter scale). Our hypothesis is that in these conditions, smooth morphotype cells swarmed slower but for a longer time than rough ones.

After 6 h on 0.3 % agar, the visible expansion of the rough morphotype was significantly higher (almost twice) than the smooth expansion. After 24 h on 0.3 % agar, both morphotypes reached the dish borders but exhibited different patterns. In the **rough** morphotype, the mother colony was branched and surrounded by an irregular colonization area. Once again, it exhibited multiple migration phases but there was an increase of the phase duration compared to the 0.6 % agar, due to the lower agar concentration (Tasaki et al., 2017). New colonies were visible around the mother colony but not connected to it, illustrating the existence of a subpopulation of cells able to swim through the 0.3 % agar. However, these swimming cells seemed less numerous than the cells in the mother colony considering their visible proportion. In the **smooth** morphotype, a predominant swimming behavior within the agar was visible, leaving a large uncovered area around the mother colony that did not display a complex colony organization like the rough. Small size of the mother colony could indicate an important share of cells leaving the mother colony to swim through the dish and forming this large EPS ring near the borders.

As explained previously, microbiologists often extrapolate colonies expansion rates to swarming and swimming rates. Therefore, both morphotypes were considered mobile as they were able to reach the dish borders within 24 h when the agar concentration was the lowest (0.3 %). Few studies can be found in the literature to compare *B. amyloliquefaciens* L-17 motility with other wild type strains of *B. amyloliquefaciens.* Measured swarming colonization rates range from 1.3 to over 1.9 mm/h (Feng et al., 2022; Liu et al., 2010; Lü et al., 2024). In swimming assays, colonization rates are comprised between 0.9 and 1.5 mm/h (Lü et al., 2024; Yang et al., 2018) with even a highly mobile strain able to expand at 14 mm/h under optimal controlled conditions (Zhao et al., 2016). Surprisingly, rates reported in literature are higher in swarming assays than in swimming assays. However, in these studies, different parameters are used (incubation time and temperature, inoculum volume, *B. amyloliquefaciens* strain, agar percentage, medium composition) and each of them can impact the bacterial motility. Therefore, they can only be used as a general comparison. **Swarming** literature values are relatively close to those from the rough morphotype (1.3 ± 0.2 mm/h at 6 h; 1.5 ± 0.3 mm/h at 24 h) or from the smooth morphotype (1.1 ± 0.1 mm/h at 6 h; >2.4 mm/h at 24 h). On the other hand, both morphotypes exhibited slightly higher **swimming** motility compared to the literature values: 2.8 ± 0.1 mm/h at 6 h; >2.4 mm/h at 24 h for the rough and 1.7 ± 0.1 mm/h at 6 h; >2.4 mm/h at 24 h for the smooth.

An obvious link could be established between the expansive behavior of colonies and the production of surfactants. Indeed, recent investigations have highlighted the role of surfactant production in enhancing the colonization of semi-solid media by *B. subtilis* (Be’er and Ariel, 2019; Dergham et al., 2021; Tasaki et al., 2017) and *B. amyloliquefaciens* (Feng et al., 2022). Although surfactant production is not strictly essential for surface colonization (Thérien et al., 2020), variations in its regulation may partly explain the differences in colony expansion observed between morphotypes. Among the surfactants, surfactin, a lipopeptide, is the most extensively studied within the *Bacillus* genus (Qiao et al., 2024; Zhang et al., 2022) and it is also produced by *B. amyloliquefaciens* strains (Feng et al., 2022; Liu et al., 2023). Rahman et al. (2021) demonstrated that surfactin significantly influences *B. subtilis* subpopulations by enhancing matrix production and facilitating the swarming of motile cells. Other secreted molecules, such as extracellular proteases and other lipopeptides (e.g., fengycin), can also contribute to this process (Ambrico et al., 2019; Dergham et al., 2021). Furthermore, Ambrico et al. (2019) associated the production of another lipopeptide, iturin, with the rough morphotypes of *B. subtilis*. Therefore, the hypothesis of a difference in lipopeptide production dynamics is proposed. The rough morphotype might exhibit early and abundant lipopeptide production, leading to rapid colonization and substantial matrix production. In contrast, the smooth morphotype might continue to colonize the agar at a slower rate.

Motility and biofilm formation are interconnected processes extensively characterized in *B. subtilis* (Dergham et al., 2021; Guttenplan and Kearns, 2013; Sanchez-Vizuete et al., 2022). Notably, the ability of cells to swim has been demonstrated to influence pellicle formation in *B. subtilis* (Hölscher et al., 2015; Liang et al., 2010). However, this relationship remains unexplored in *B. amyloliquefaciens*. To address this gap, we monitored the stages of pellicle production to ascertain whether variations in motility among different morphotypes affect pellicle formation.

### 3.2 Pellicle morphogenesis

**Fig. 3:**
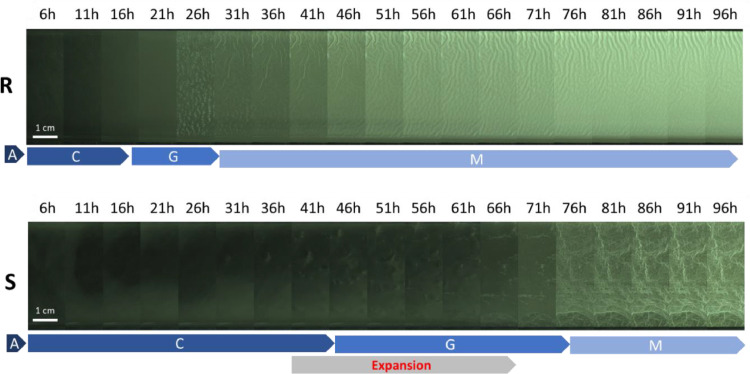
Sequenced morphogenesis of pellicles for 4 days. Pellicles have been incubated at 23 °C in GMM. Steps of the pellicle formation as defined by Lee et al. (2019) are represented beneath the photos A: attachment, C: Conversion, G: growth, M: maturation. Scale bar is 1 cm.

Pellicles’ morphogenesis has been monitored for both morphotypes in liquid GMM (Fig. 3). The whole movie recorded at 4 frames per hour is available in **Suppl. Data A Movie S1**.

**Fig. 4:**
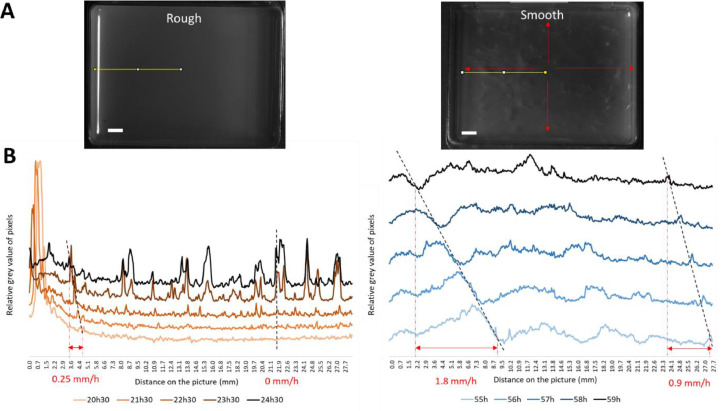
Rough (left) and smooth (right) pellicles during the expansion phase detected in Movie S1. (A) Top view of the dish and localization of the image analysis (yellow line). Red arrows show outward expansion movement. Scale bar is 5 mm. (B) Grayscale profiles along the yellow line section, from the center to the edge of the pellicle.

**Fig. 5:**
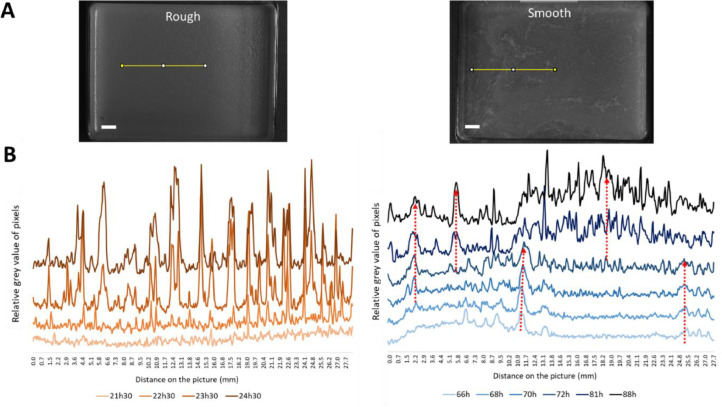
Rough (left) and smooth (right) pellicles during wrinkles and folds formation. (A) Top view of the dish and localisation of the image analysis (yellow line). Scale bar is 5 mm. (B) Grayscale profiles along the yellow line section. Dotted red arrows show the heterogeneity in wrinkles emergence (in time and space) with the smooth morphotype.

During the initial hours of growth, both morphotypes displayed a heterogeneous distribution across the dish, with active swimming behavior clearly visible (**Movie S1**). Bacteria in suspension formed collective rolls, as documented by Lee et al. (2019). After approximately 16–18 h, collective cells motion of the **rough** morphotype became indistinguishable due to pellicle formation at the surface. Pellicle production was a coordinated action, occurring simultaneously and uniformly across the dish. From 18 to 27 h, pellicle moved toward the dish walls at a maximum speed of 0.25 mm/h near the edges, as detailed in Fig. 4, likely resulting from matrix production. During this phase, pellicle’s surface became granular (22 h) and regular wrinkles appeared (22–24 h), as shown in Fig. 5. Light reflection on the liquid medium added a bias on the dish border before pellicle formation (Fig. 4**B**). It disappeared with pellicle formation. The pellicle increasingly scattered light throughout the experiment, indicating a thickening process until the end of the experiment. By the experiment’s conclusion, at 96 h, the pellicle had thickened and was characterized by a homogeneous white surface with wrinkles (Fig. 3). This morphogenesis aligns with the five stages of pellicle formation identified in *B. subtilis* by Kobayashi (2007) and Lee et al. (2019): attachment, conversion, growth, maturation and death. The first four stages were entirely transposable to the pellicle formation of *B. amyloliquefaciens* L-17, with the rough morphotype’s timing mirroring the observations of Lee et al. (2019).

However, the timing and final appearance of the pellicle differed for the **smooth** morphotype. Initially, collective cells motion was visible for 41 h, which was twice as long as observed for the rough morphotype. Then the pellicle underwent an important expansion movement for almost 30 h (from 41 h to 69 h) that ended with pellicle thickening (Fig. 3). During this period, microcolonies appeared, widened and eventually joined; they moved rapidly outwards: 0.9 mm/h at the center of the dish and 1.8 mm/h near the edges (Fig. 4). These important expansion rates were equivalent to the results of the swimming assay. During this expansion phase, smooth pellicle seemed to climb on the dish walls as previously described by different authors (Hölscher et al., 2015; Krajnc et al., 2022; Lee et al., 2019). Maturation phase occurred from 76 h to 96 h, characterized by a thickened pellicle with pronounced wrinkles, distinct from the rough morphotype. The final pellicle of the smooth morphotype appeared thinner and more heterogeneous compared to the rough one, and it lost its mucous appearance observed on agar plates. This change might be attributed to the incubation conditions (uncovered dishes) that resulted in a drier environment.

Morphogenesis timing and final morphotypes seem correlated with *B. amyloliquefaciens* L-17 behavior on 0.6 % agar dishes. In both scenarios, the rough morphotype rapidly colonized the medium, producing a robust biofilm, whereas the smooth morphotype continued to colonize the medium but formed a thinner pellicle. Several hypotheses could explain this delay in pellicle formation. First, inhibited cell motility might prevent bacteria from reaching the surface. However, swarming and swimming tests suggested the motility of both morphotypes and collective rolls observed in liquid GMM further supported this. If cells can indeed reach the surface, it suggests that the delay is due to a regulatory mechanism governing pellicle formation. Transition from motile cells to matrix-producing cells has been well described in *B. subtilis* (Guttenplan and Kearns, 2013; Kröber et al., 2016; Steinberg et al., 2020; Tasaki et al., 2017; Yang et al., 2018; Zhao et al., 2016, 2015) and a motile behavior is often opposed to a matrix production behavior (Charron‐Lamoureux et al., 2024). Sanchez-Vizuete et al. (2022) showed the role of oxygen depletion on motile cell migration toward the medium surface to produce EPS. The smooth morphotype could be less sensible to oxygen depletion or bioconvection could enrich the medium with oxygen, as described by Jánosi et al. (1998). Finally, this delay could also be due to attachment difficulties, inhibition of matrix production genes or quorum sensing regulation. Indeed, on one hand the rapid and coordinated biofilm production of the rough morphotype, and one the other hand the slow and heterogeneous EPS production by the smooth morphotype suggest an implication of the quorum sensing system.

As for the final aspect of pellicles, a previous study showed that addition of surfactant (surfactin or an exogenous molecule such as Tween20) allows a rapid and homogeneous colonization of the air-liquid interface and at the same time reduces pellicles elasticity (Rühs et al., 2013). This description fits the rough pellicle morphogenesis, as well as its expansion rate on agar. At the contrary, a late production of a small amount of surfactant by the smooth morphotype would explain its slower and heterogeneous colonization of surfaces.

On the final structural aspect of biofilms, the smooth pellicle presented numerous disordered wrinkles and folds when the rough pellicle seemed flatter (Fig. 3**, Suppl. Data B**). Few early large vertical folds, followed by small numerous disordered wrinkles, emerged from the flat smooth pellicle, the number of visible wrinkles increasing progressively with time. At the end, many vertical hierarchical structures fully covered the mature smooth pellicles. In contrast, as shown in Fig. 5, vertical periodic undulations emerged from the flat rough pellicle quasi- simultaneously within a relatively short period of time (less than one hour). They became regularly spaced (2.0 ± 0.4 mm), as expected for wrinkling process in material science. Some of the wrinkles begun to be more pronounced while others disappeared with time. In previous studies (Dervaux et al., 2009; Pocivavsek et al., 2008; Rodriguez et al., 1994; Trejo et al., 2013), the millimeter wavelength of the wrinkles observed in pellicles of *B. subtilis* NCIB 3610 was explained using a mechanical approach based on pellicles elasticity: solid-like and elastic pellicles grow on top of liquid within a limited and confined space. This constrained growth leads to an in-plane compression (a compressive force within the pellicle) which progressively increases with time up to a given critical threshold. At this threshold, an out-of-plane deformation becomes more favorable energetically and the buckling instability occurs. Wrinkles start to emerge with a specific wavelength that depends on physical parameters like elasticity, thickness and gravity and then follow folds which concentrate all the deformations. Thus, in the present study, the spatial organization of cells inside the pellicle, the growth rate or the matrix production must have varied between morphotypes to form this very different final aspect of the pellicles (Fig. 3**, Suppl. Data B**). In Fig. 3 and in **Suppl. Data B**, additional wrinkles perpendicular to the dish edges are clearly visible on rough pellicles. Trejo et al. (2013) explained these wrinkles by pellicles climbing to the edges and by elasto-capillary effects.

### 3.3 Mechanical features of pellicles

In the following section, we considered the mature pellicles and explored their elastic behavior as a two-dimensional material.

#### 3.3.1 Mechanics of pellicles: deformability

**Fig. 6:**
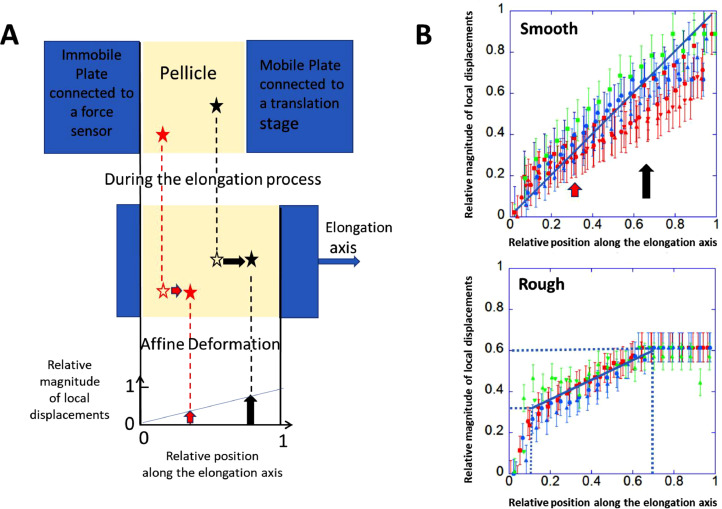
Uniaxial Elongation: A (top) Top-view schematic diagram of the experimental setup. Small portions (illustrated by stars) can be visualized by a camera placed above the pellicle. A. (middle) Plate displacement is recorded by filming the elongation process. A. (bottom) A linear variation of local displacements with position is expected when stretching a homogeneous and elastic material along one axis. B. Local displacements as detected by the PIV analyses on smooth (top) and rough (bottom) pellicles. Spatial position along the elongation axis is relative to two positions: immobile plate (= 0) and mobile plate (=1). Blue line illustrates an affine deformation. Error bars correspond to one pixel value, on both sides of each data point. Each color represents one sample repetition (*n* = 3) and each symbol represents a PIV analysis performed on different pairs of images taken at different elongation values.

To quantify the mechanical properties of pellicles, we used the elongation setup described in Trejo et al. (2013). As marked by two black and red stars in Fig. 6**A**, two distinct parts of the pellicle are expected to move a certain distance during the elongation process; their final position depends on their initial location. Arrows indicate the displacement vectors. Close to the mobile plate (right side), the material should displace at the highest extent (longest arrow) while, close to the immobile plate (left side), the extension should be almost null (shortest arrow). Hence, at any positions, a linear (affine) variation of the displacement magnitude, the blue line in Fig. 6**A** (bottom), is expected for a homogeneous and elastic material. This variation indicates how the material is deforming.

The relative magnitude of local displacements in the elongation direction is plotted in Fig. 6**B**. For each type of pellicle, the same displacements were detected between repetitions, indicative of reproducibility. Surprisingly, smooth pellicles deformed quasi-uniformly while morphological non-uniformities were present during their genesis. Far from the edges, in bulk, regions of different white greyscale deformed in the same way. Hence identical deformability indicates an identical stiffness of these non-uniform morphological regions. Only very slight deviations from linearity (first bisector, blue line) could be detected close to the mobile plate (right side). This effect was previously observed on *B. subtilis* pellicles (Hollenbeck et al., 2016) where additional measurements with and without meniscus allowed the authors to conclude that the pellicle structure near attachment surfaces was different than the rest of the pellicle. The central region of the pellicle was stiffer and conversely, the portion of the pellicle near the attachment surface was softer. As observed in the present samples, higher variations in local displacements indicated higher deformations near the edges.

For rough pellicles, deformation profile in Fig. 6**B** exhibited marked deviations from linearity and non-uniformity, which was rather unexpected as rough pellicles exhibited uniform morphological features. Same deviations could be detected whatever the pellicle was. In the interval [0, 0.15] of relative position, near the left edge (immobile plate), local displacements reached up to 0.33 (in relative magnitude), indicating that matter moved up to one-third of the total elongation (about 0.2 mm). Therefore, near this edge, pellicles were highly deformable and soft compared to the next juxtaposed central region. In the central part, within the interval [0.15, 0.70] in relative position, displacements followed another linear relationship with the positions; this central part extended therefore over the largest areas distant by 2 mm from the immobile plate and by 4 mm from the mobile plate. Linear fit of displacements data indicated that matter elongated by another one-third in magnitude and more precisely by 27 %. In the interval [0.70, 0.95], i.e.*,* in regions that were close to the mobile plate, PIV results showed displacements of constant magnitude equal to 0.60 (in relative unit). All the matter moved by the same magnitude meaning that this region just translated without any deformation. Therefore, these pellicle areas were significantly stiffer than the rest. Then, in the last interval [0.95, 1], the mobile plate moved to 1 (in relative magnitude) while nearby pellicles only moved by 0.6; a deep, quasi-discontinuous step in displacements occurred within this narrow range. A detailed and careful analysis is presented in **Suppl. Data C** (Detailed analysis of local displacements of a rough pellicle near the mobile plate). This detailed analysis confirmed the presence of a very narrow and easily deformable area, juxtaposing the firmly attached biofilm to the mobile plate. As a result, when the mobile plate was translated by 0.6 mm, the very nearby narrow region elongated by 0.24 mm while the other parts of the pellicles elongated by 0.36 mm. In this scenario, the central deformable part only deformed by 0.16 mm, a value much lower than the applied elongation.

Note that we have neglected the presence of vertical structures such as wrinkles and folds in the deformation analysis to simplify the discussion. In principle, pulling horizontally on a vertical wrinkle or a fold should simply flatten it out and produce displacements of vertical matter towards the horizontal plane, without deformation of the inner structure. This effect should lead to additional horizontal area of matter that should enlarge when progressively flattening the fold. This enlargement was not detectable on the different pairs of images taken at different elongation values. This suggests that the vertical structures were not unfoldable and firmly connected to the horizontal structures.

#### 3.3.2 Mechanics of pellicles: forces

**Fig. 7:**
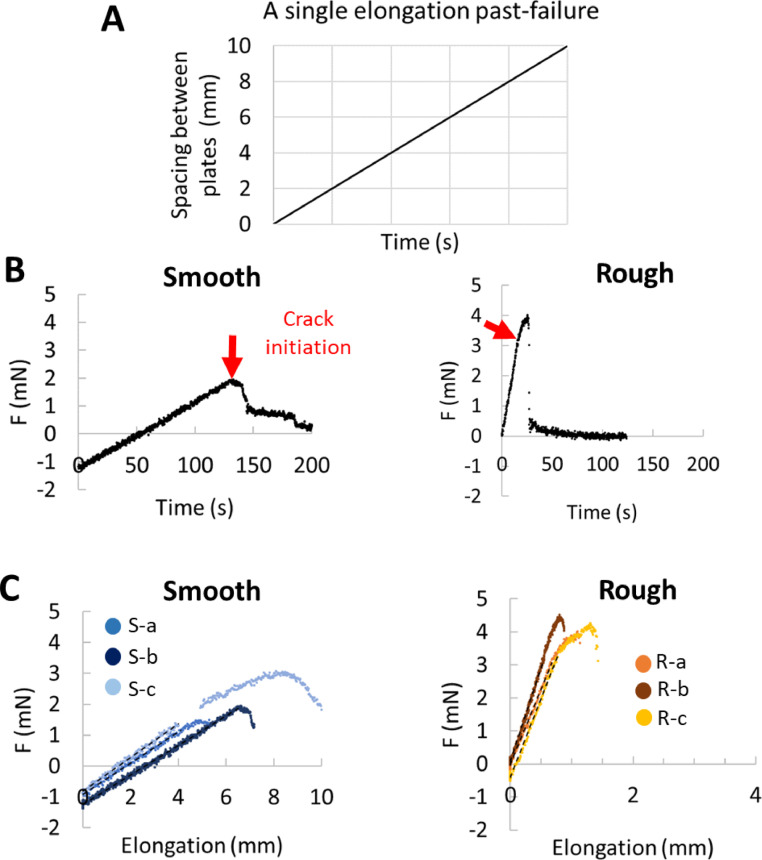
Mechanical Force measurements. (A) A typical single elongation past-failure experiment, where the mobile plate moved at constant speed over time (top) and elongated the pellicles. (B) two elongated smooth (left) and rough (right) pellicles transmitted a mechanical force to the force sensor. The mechanical force was measured over time past failure. Red arrows show crack initiation. (C) Elongation-Force curve measured on three (smooth, blue symbols, left; rough, orange-brown symbols, right-hand side) samples of each. We consider the elongation of the entire pellicles, for the smooth samples and of only the pellicles central part (for rough samples). Data are fitted linearly to extract the pellicles stiffness in the elastic regime (black dashed line).

All these deformations generated a stress or a mechanical force within the pellicles that depended on their stiffness. We were able to measure the force transmitted by the pellicle to a dedicated force sensor during its elongation (see Fig. 6**A**). Fig. 7 illustrates typical measurements during a single elongation past-failure. The mobile plate moved away from the immobile plate at a constant speed during the force measurements (Fig. 7**A**). Fig. 7**B** shows two representative force curves that were recorded on one smooth (left) and rough (right) pellicle during the plate translation. The curves show a first linear variation with time followed by a sudden decrease due to pellicles failure. Past failure, pellicles did not transmit mechanical forces anymore as they released the stress due to elongation. In Fig. 7**B**, both curves presented different features: initial force value, slope and range before pellicles failed.

Curves recorded for **smooth** pellicles (Fig. 7**C, left**) presented many similarities with the curves previously recorded for *B. subtilis* pellicles (Hollenbeck et al., 2016). The −1 mN intercept value (−1.2 ± 0.2 mN, on average) was negative suggesting the existence of a residual compressive force due to growth in a confined space (Douarche et al., 2015). This is not surprising considering the important expansion of the pellicle during its formation, observed in **Movie S1** and quantified in Fig. 4. Release of this force induced an expansion of the pellicle, as observed past failure or past detachment to the surfaces without confinement effects (not shown); lateral dimensions of pellicle increased to the detriment of its thickness (thinning process) (Douarche et al., 2015). Then temporal linear variation at a constant speed suggested an elastic response of the entire smooth pellicle. As seen previously from the weakly detectable difference in the deformation profile in Fig. 6, only regions near the plates experienced a slightly larger elongation than the central region, a small effect that can be neglected in the following. Force-elongation curves recorded for three different smooth pellicles are presented in Fig. 7**C**. All the curves showed a well-marked linear dependence, of similar slopes, that extended up to a maximum value (failure) which varied from pellicles to another. Being equal to the slope value (dashed line), stiffness of smooth pellicles was found equal to 0.51 ± 0.04 N/m on average. Consequently, smooth pellicles of *B. amyloliquefaciens* and *B. subtilis* had very similar stiffness (0.51 ± 0.04 N/m (this study) and 0.31 ± 0.06 N/m (Trejo et al., 2013), respectively) and identical residual compressive force (−1.2 mN for both). In other words, they shared very similar physical properties. Interestingly, both pellicles presented a wrinkle morphotype and needed a few days to form (in Trejo et al. (2013), the pellicle was wrinkled at 67 h).

By inspecting the curve recorded on a **rough** pellicle in Fig. 7**C**, it became obvious that the intercept value was nearly zero (−0.15 ± 0.25 on average) and the elongation magnitude before failure was much lower for rough as compared to the smooth pellicle. No residual compressive force was therefore detected which is quite surprising as pellicles exhibited vertical structures like wrinkles which are associated with growth (compression under confinement) and elasticity effects. We suspect that this lack of initial compression is related to the different steps noticed during its morphogenesis in our experimental conditions. As discussed earlier, the pellicle started to appear 16 h after the inoculation and to present early wrinkles. Then, instead of continuing to expand laterally, instead of enlarging the wrinkles vertical level and generating large folds during its growth, the pellicle thickened. We can therefore hypothesize that vertical thickening, which followed the early wrinkling process (22–24 h), progressively has reduced lateral compressive force magnitude up to a non-detectable value at 72 h. Previous analysis on deformability indicated the composite nature of pellicles which are juxtaposed regions of different deformability. As discussed previously, the central region, that covered the largest area, only deformed up to 27 % of the total elongation. Therefore, the applied elongation value, presented in Fig. 7**C**, has been corrected and corresponds to a true strain value, e.g., to a true elongation of the central part equal to 1 mm when the plate moved by 3.7 mm. Here again, a linear dependence of the force-elongation curves could be observed from the experimental data measured on the three rough pellicles, as expected for elastic materials.

The averaged stiffness value of rough pellicles was found equal to 5.1 ± 0.6 N/m. This value corresponds to the stiffness of only one part of the rough pellicle, a central part that extends over the relative position 0.15 to 0.70. In other words, size of this portion would be (0.70–0.15) = 0.55, i.e.*,* about half of the pellicle length. By definition, stiffness of a material depends on its shape, its dimensions, its constitutive elements and on inherent properties of the constituent material itself. The shorter the material, the higher its stiffness. Hence a full-length pellicle would have a stiffness equal to 2.5 N/m which is about five times higher than the stiffness of smooth pellicles (0.51 N/m). As shown previously in the deformability analysis, portion of rough pellicles located in the interval [0.70–0.95] might be even stiffer than that, as its deformation was not detected by PIV. We can estimate that a full-length pellicle made up of this portion would have a stiffness equal or higher than 25 N/m.

A last feature on failure can be extracted from the mechanical force experiments. Smooth pellicles were able to elongate almost 40 % of their initial length (5 mm over 13–14 mm) before failure while rough pellicles disrupted quite rapidly. In the latter case, disruption occurred mostly very near the attachment sites on the plates, where the soft parts were severely deformed, as illustrated in **Suppl. Data D**.

As considered by Trejo et al. (2013), the presence of wrinkles and folds is as a direct consequence of pellicles’ elasticity, which can be driven by EPS composition or cells organization inside the biofilm. In *B. subtilis*, the wrinkles have been attributed to the expression of several genes encoding matrix components (*tasA, bslA, epsA-O*) (Arnaouteli et al., 2023; Mammeri et al., 2019; Trejo et al., 2013). However, this contradicts the analysis by Morris et al. (2024) who found that the amphiphile protein BslA enhanced pellicle elasticity, whereas TasA reduced it. An orthologue of BslA has been identified in *B. amyloliquefaciens* FZB42, where it was responsible for the wrinkled morphotype of colonies and conferred hydrophobic properties (Morris et al., 2017). Therefore, it is possible that both BslA and TasA are responsible for the wrinkled morphotype, but only BslA is positively correlated with pellicle elasticity.

## 4 Conclusion and perspectives

Although studies on the mechanisms of biofilm formation in *B. subtilis* have been widely explored, *B. amyloliquefaciens* has been less studied, while it is of increasing interest to the scientific community due to the wide range of applications of this microorganism in various fields (agri-food, soil, agronomy, etc.). In this work, we were particularly interested in *B. amyloliquefaciens* strain L-17, which produced two phenotypic variants. The existence of these two distinct morphotypes from the same strain is an opportunity, as they naturally allow comparison. In addition, it would give us a better understanding of the diversity of behavior that the same strain can display.

Within 24 h, **rough** morphotype formed a visually homogeneous and wrinkled pellicle with high stiffness. Surprisingly, this mechanic stiffness was not homogeneously distributed on the pellicle. This non-uniform response to mechanical load remains unexplained as the rough pellicles appeared homogeneous and needs further investigations to be clarified. A very different behavior was observed with the **smooth** morphotype. Its expansion on agar medium was slower than the rough morphotype. In the same way, it needed more time to produce a biofilm at the air-liquid interface. The result was a heterogeneous and wrinkled pellicle with high elasticity and high residual compressive force. The hypothesis was proposed that the rough morphotype rapidly secreted lipopeptides, as it could explain both its fast colonization of surfaces (agar and air-liquid interface) and its homogeneous pellicle formation. For the first time, it has been shown that the morphogenesis model of pellicle formation by *B. subtilis* was applicable to *B. amyloliquefaciens*.

To extend understanding of the mechanical properties of an air-liquid interface biofilm, which represents a complex viscoelastic material, it will be interesting to assess the mechanical strength of the pellicle more deeply, using a combination of techniques such as interfacial rheology and confocal laser scanning microscopy. Indeed, the distribution of subpopulations within the biofilm during growth is a crucial element not to be overlooked, as highlighted in this article through expansion assays on agar medium. Moreover, it is now recognized that EPS plays an active role in the functional properties and social interactions within biofilms, such as signaling, genetic exchange, the creation of microcosms and mechanical stability. The matrix is constantly produced and dynamically changed, affecting structure and function throughout the pellicle life cycle. The dynamic biochemical composition of the pellicle must therefore to be explored, with the extraction and characterization of numerous matrix components, or by using molecular tools such as transcriptomics and mutagenesis. Particular attention could be paid to 1) genes responsible in the EPS matrix production such as *tasA, bslA, epsA-O*; 2) genes involved in motility, especially *hag* gene of flagellin; 3) genes linked to metabolism such as oxygen detection (*hemAT, fnr)*, production of surfactin (*srf)* or quorum sensing. This has already been done in *B. subtilis* but has yet to be applied to *B. amyloliquefaciens*.

## Funding

This work was supported by a fellowship from Occitanie Regional Council [Grant n°00089602 / 21013062], Institut Universitaire de Technologie (IUT) de Toulouse Auch Castres, Toulouse University, Auch and Gers Local Councils.

## Declaration of competing interest

The authors declare that they have no known competing financial interests or personal relationships that could have appeared to influence the work reported in this paper.

## Data Availability

The authors are unable or have chosen not to specify which data has been used.

## Appendix Supplementary materials

Image, application 1

Image, video 2
