# Supplementary material for: Morphogenesis and mechanical properties of Bacillus amyloliquefaciens biofilms: a comparative study of rough and smooth morphotypes
Source: Curr Res Microb Sci. 2025 May 10;8:100403. doi: 10.1016/j.crmicr.2025.100403 (PMC12141842; doi:10.1016/j.crmicr.2025.100403)
Supplement: Supplementary file 1 [file mmc1.docx]

# **Appendix A. Supplementary data**

## A. Morphogenesis movie of rough and smooth pellicles

**Movie S1 :** Movie of pellicles morphogenesis from 6 h to 96 h in GMM at 23 °C. Top: smooth; bottom: rough. A picture is taken every 15 min. Dish dimensions = 4.6 x 6.2 cm.

## B. Final morphotype of *B. amyloliquefaciens* L-17 pellicle after 96 h in GMM at 23 °C.


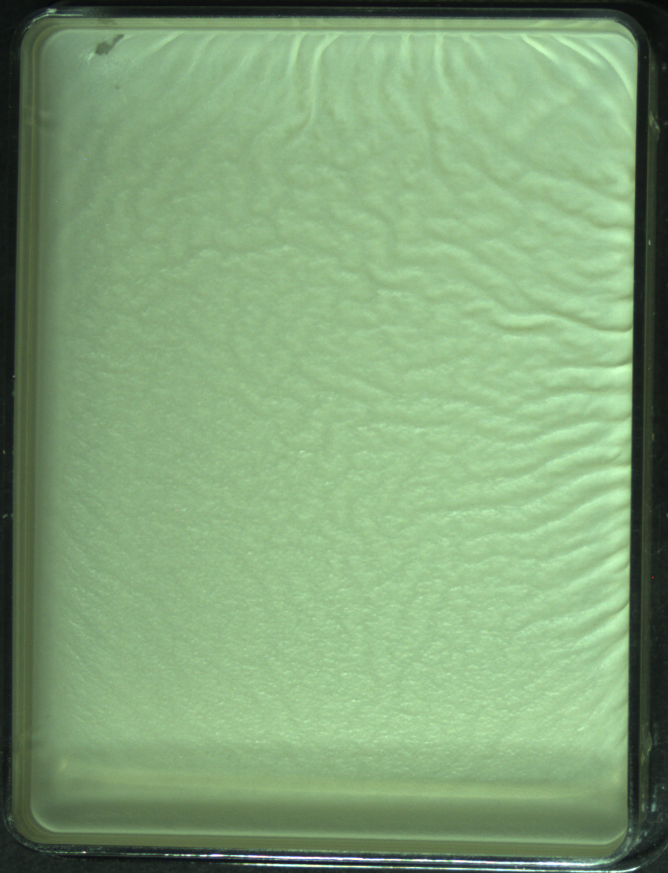

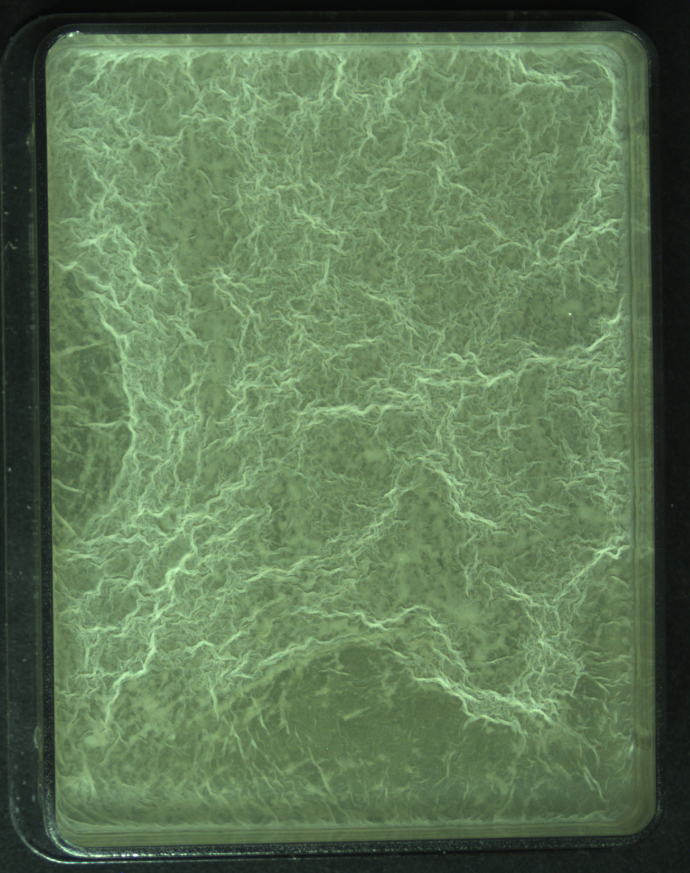


**Supplementary Figure 1**: Photo of the final aspect of rough (left) and smooth (right) pellicles after 96 h of incubation in GMM at 23 °C. Dish dimensions = 4.6 x 6.2 cm.

## C. Detailed analysis of local displacements of a rough pellicle near the mobile plate

We used the ImageJ PIV plugin to detect local motions [39,40]. A pair of images (taken at two successive instants) is sliced into small regions called interrogation windows, from which correlations are automatically detected. Each data point in **Fig. 6** describes PIV results in one region, centered at a given position. **Suppl. Fig. S2** corresponds to a copy of **Fig. 6** on which two reference points (empty symbols) located at the two reference positions 0 and 1 have been added. The 0 relative position refers to pellicle edge at the position of the immobile plate connected to the force sensor and 1 to pellicle edge sticked to the mobile plate which is connected to the translation stage. We further consider that pellicle edges firmly and intimately sticked to the plates and that they followed the plate motion without any deformation. Therefore, relative magnitudes of pellicle displacements are respectively 0 and 1 at the two reference positions, leading to their two (0,0) and (1,1) reference coordinates.


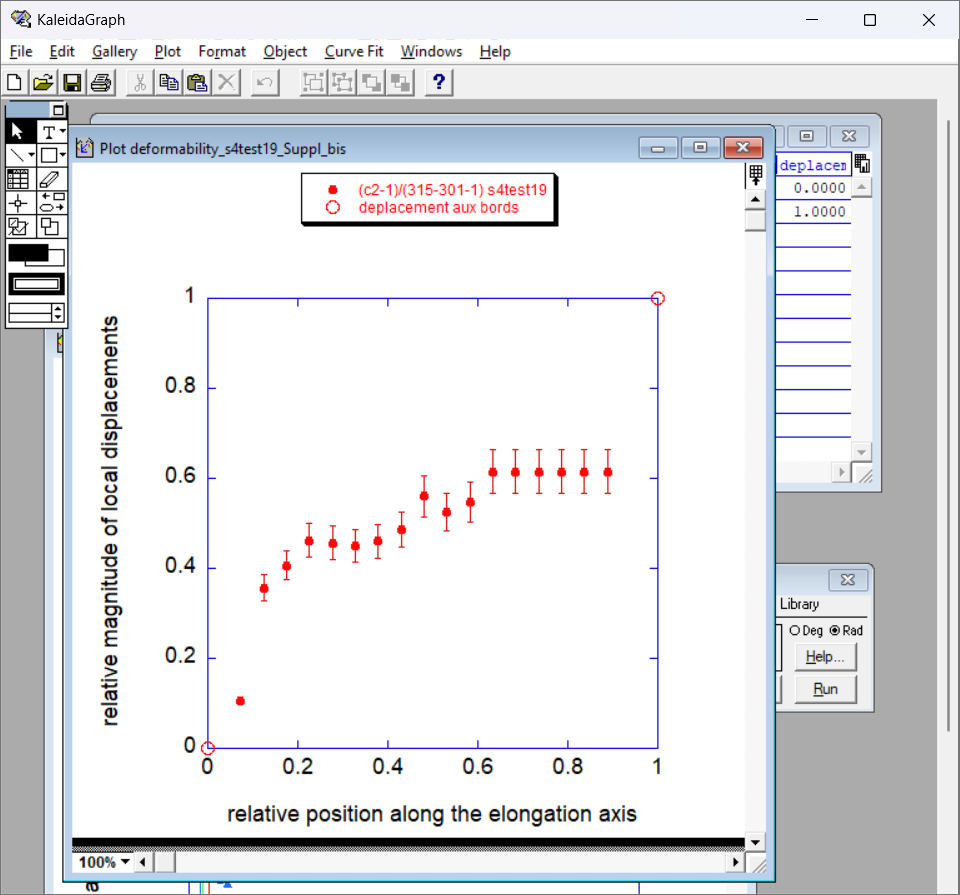


**Supplementary Figure 2**: Deformability of a rough pellicle as revealed by PIV results. Local displacements of small regions are detected by PIV analysis and are plotted versus the position of each region center along the elongation axis (red full-filled circled symbols). Error bars are estimated assuming an uncertainty of one pixel in detected displacements. The two empty symbols denote the position of the two reference positions; their (0,0) and (1,1) coordinates stand for the two extreme regions, firmly attached to the two plates without deformation (0,0) for the immobile plate and (1,1) for the mobile plate. Solid lines illustrate two steps.

**Suppl. Fig. S2** clearly shows the presence of two steps connecting the central part to the two reference positions. A smooth step is visible near the immobile plate and a deep (discontinuous-like) one near the mobile plate, indicating the existence of soft and more pliable regions nearby the two plates. In the following text, we examine in detail the pair of images from which PIV results have been extracted in order to understand steps, more specifically the deep step on the translated side. **Suppl.** **Fig. S3A** shows two images (converted to 8-bit type) of a rough pellicle at two successive instants when initial pellicle begun to be elongated by a translation motion from 0 to about 0.6 mm. On the left-hand side, pellicle is attached to the immobile plate and pellicle’s edge is clearly visible, contrasted, on both images as pellicle scattered light making them brighter than the plate itself. A thin, straight, dark zone, delimiting the position of the plate-biofilm junction, can be easily detected with the naked eye. A black dashed line indicates this position on both images in **Suppl. Fig. S3A**. On the right-hand side, pellicle is attached to the mobile plate and its edge is less visible, less contrasted than the other. Its edge position (indicated by a dark straight line) shifts to the right side during the elongation process, *i.e.* during the translation, as marked by the arrow between the two images.

**Suppl. Fig. S3B** shows a magnification of the two blue areas together with a detailed analysis of the plate position at the two successive instants. We examine grey values along a thin orange line perpendicular to the plate in order to precisely and quantitively determine plate’s position from the plot profile. As suspected, the dark region (low grey value) contrasts with the surrounding brighter region (high grey value). Precise analysis, performed on the pair of images, shows a slight shift by one-pixel of the plate position or equivalently by 0.046 mm (to be compared to the 0.64 mm-shift). As expected, this plate can be considered as immobile.


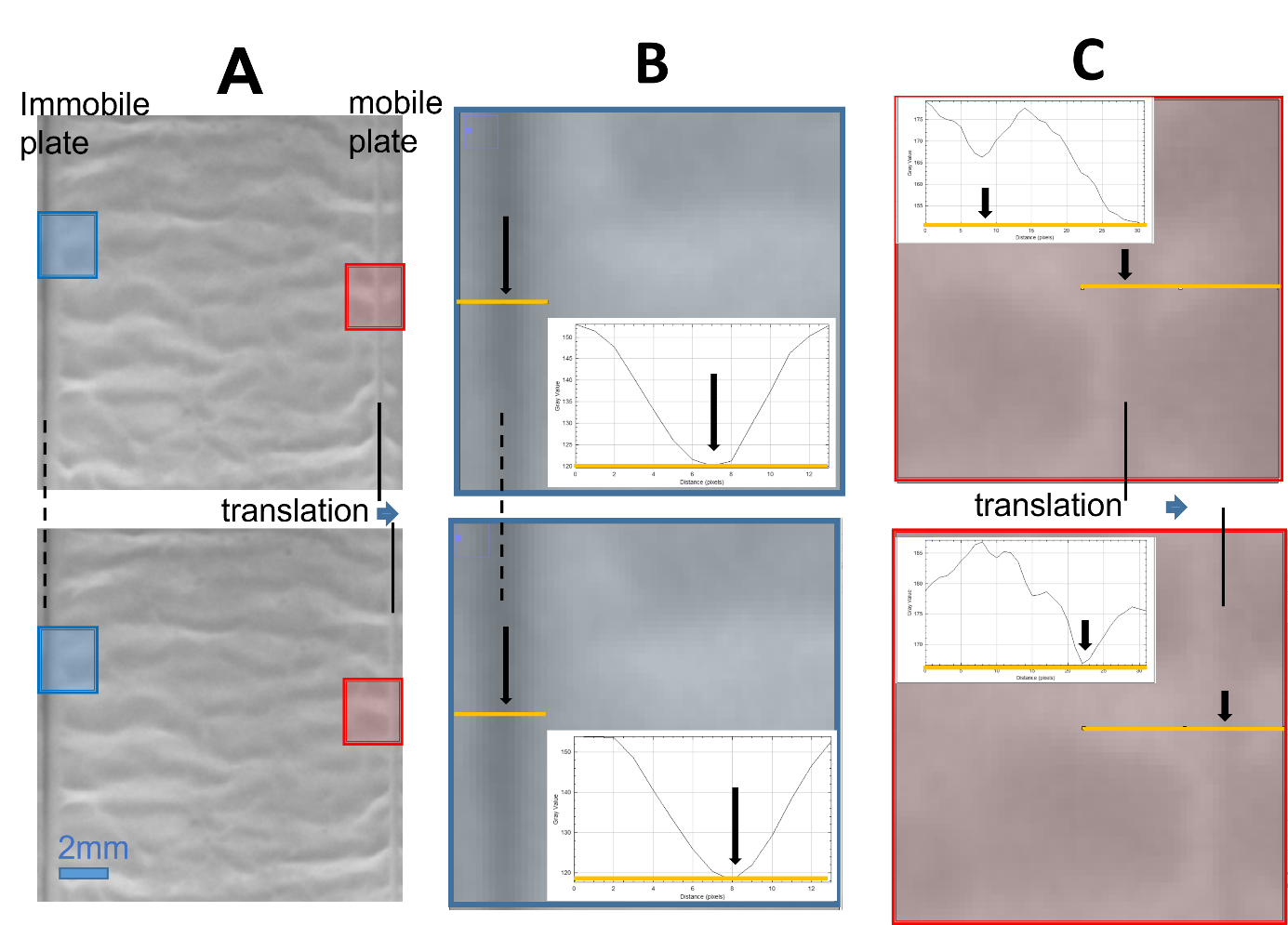


**Supplementary Figure 3:** **A.** Magnified images of a rough pellicle before and after an elongation from 0 to 0.6 mm. Location of the two plates is indicated by two black lines (dashed for the immobile one and solid for the mobile one). Further detailed analyses of grey values performed near the immobile plate (blue area, **B**) and near the mobile one (red area, **C**) allow to monitor position of the two pellicle edges.

**Suppl. Fig. S3C** shows a magnification of the two red areas where location of the translated plate can be analysed. Pellicle edge is less visible but still detectable in the plot profiles. Local minimum in the grey values shifts towards the right-hand side by 14 pixels *i.e.* by 0.64 mm. Hence this careful analysis of the biofilm edge allows us to fix the two reference points in **Suppl. Fig. S2**. It remains to explain/check the existence of deep steps associated with more soft and pliable regions. As an example, we will focus on red areas where step in displacement should be pronounced, as compared to blue areas.

**Suppl. Fig. S4** reports a detailed analysis of grey levels along a segment perpendicular to pellicle edge. Same red areas are considered and illustrated magnified images have been contrasted for clarity reasons. The (orange) segment recovers the mobile plate (indicated by a black arrow pointing downwards), the bright area of the pellicle edge (an orange arrow pointing upwards) and a part of pellicle scattering less light and appearing darker than its surroundings. Size of this last dark area, delimited by orange arrows pointing downwards and upwards, can be estimated from the plot profile. **Suppl. Fig. S4** shows, without ambiguity, that the dark area elongates from 22 pixels to 28 pixels, meaning that pellicle, located at this dark area, near the edge, deformed up to 6 pixels; note that this deformation can be seen by visual inspection of the two images. Therefore, very soft and pliable region is adjacent to the edge sticked to the mobile plate and, if plate is translated by 14 pixels, pellicle’s central part is only submitted to a moderated translation of 8 pixels. As a consequence, local displacements (their relative magnitude) of the central part only reaches 8 pixels (about 0.6) instead of 14 (1), as detected accurately by PIV results.


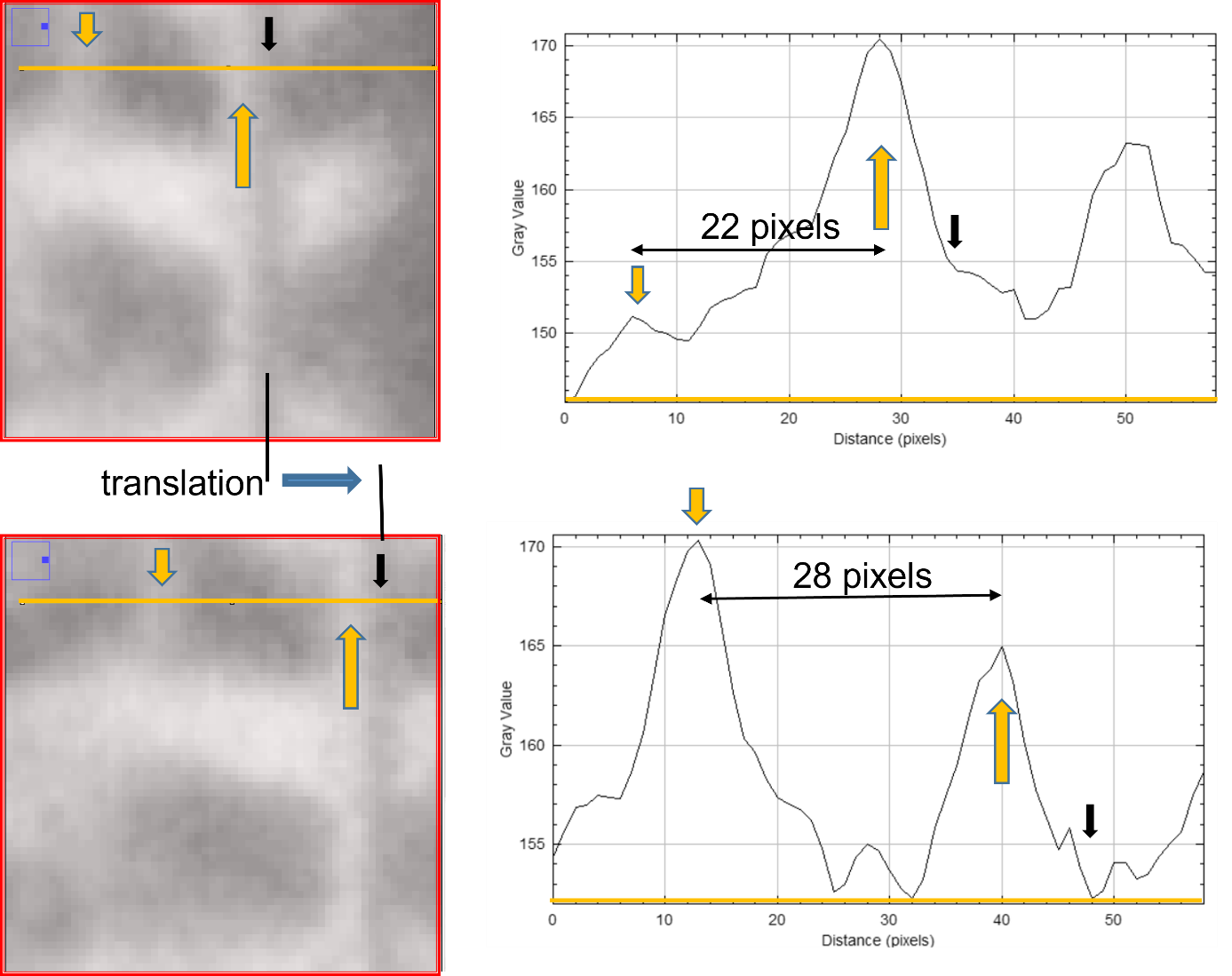


**Supplementary Figure 4**: Magnified and contrasted images of a pellicle edge and of adjacent areas. Simple visual inspection and plot profiles reveal that the dark adjacent area elongates from 22 to 28 pixels. As a consequence, the juxtaposing central part of the pellicle is only submitted to a moderated intermediate elongation value.

## D. Detached or ripped pellicles at the end of the stretching process.


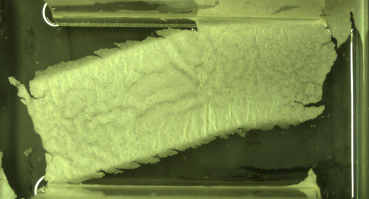

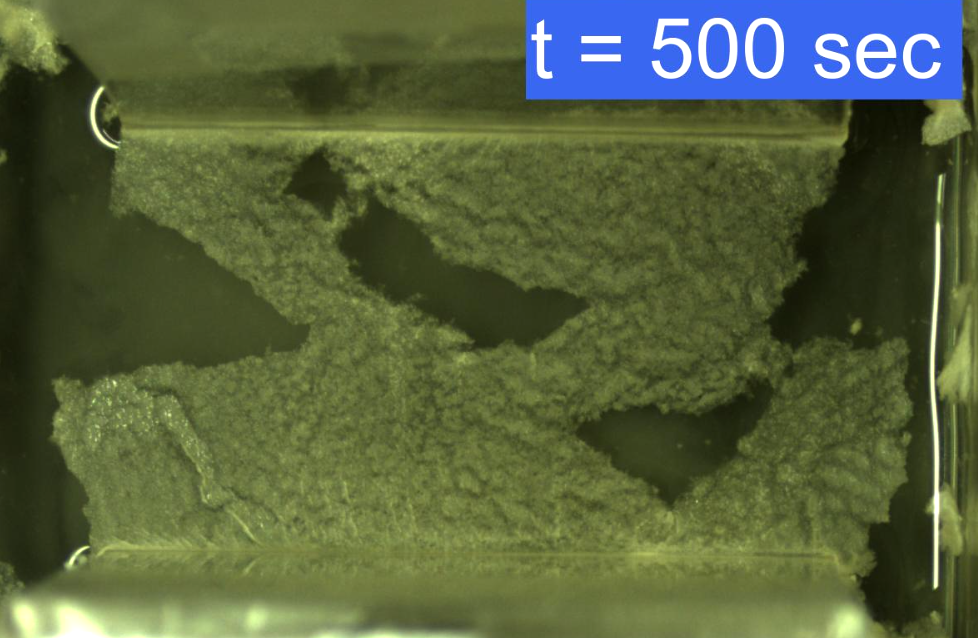


**Supplementary Figure 5***:* Detached or ripped pellicles at the end of the stretching process. Left: Rough. Right: Smooth.
